# Exploring the clinical capabilities and limitations of ChatGPT: a cautionary tale for medical applications

**DOI:** 10.1097/JS9.0000000000000523

**Published:** 2023-05-24

**Authors:** Sheng Li

**Affiliations:** Department of Anorectal Surgery, Ningbo No.2 Hospital, Ningbo, Zhejiang, People’s Republic of China

*Dear Editor*,

ChatGPT (Chat Generative Pretrained Transformer), is an artificial intelligence model developed by Open AI based on deep learning technology^1,2^. It can simulate human natural language processing ability, understand natural language, and generate language models. Since its release, ChatGPT has been widely popular and has been praised for passing the Turing test, passing a legal exam at the University of Minnesota, and achieving excellent results in another exam at the University of California, Berkeley. People have tried using it to write resumes and cover letters, explain complex topics, and provide dating advice. These performances of ChatGPT are truly remarkable, leading some to wonder if it will soon replace doctors. However, when asked about its potential applications in medicine, ChatGPT identified three major shortcomings: first, the immaturity of artificial intelligence technology, which cannot fully replace the professional knowledge and skills of doctors; second, the problem of data bias, which affects the accuracy of ChatGPT’s results if the training data is biased; and third, privacy and security concerns, as medical data is the private information of patients and its misuse or leakage can have serious consequences. Therefore, privacy and security must be carefully considered when using ChatGPT technology.

In exploring ChatGPT’s clinical capabilities, it was found that when answering basic and simple medical questions, ChatGPT can provide acceptable answers through its powerful retrieval ability. However, when it comes to more complex clinical decisions and professional knowledge, ChatGPT’s performance is unsatisfactory and even produces incorrect answers. For example, when asked how to choose an adjuvant chemotherapy regimen for stage II colon cancer with pathological staging, ChatGPT produced many errors, as shown in Figure 1. However, after multiple questions and reminders that stage II colon cancer should consider high-risk factors for recurrence (such as MSI status), ChatGPT’s answers improved, as shown in Figure 2. However, there are still many problems with its answers, such as ChatGPT’s inability to understand what pathological staging is, its lack of understanding that stage II colon cancer does not involve lymph node metastasis, and its failure to use the recommended treatment guidelines for this staging. Its answers to medical professional questions are not as good as those of IBM Watson. It is clear that ChatGPT cannot currently provide good references for clinical decision-making, let alone make autonomous decisions. However, from the different answers before and after, we can see that ChatGPT has some learning ability and can improve the quality of its answers through training with human conversation.

**Figure 1:**
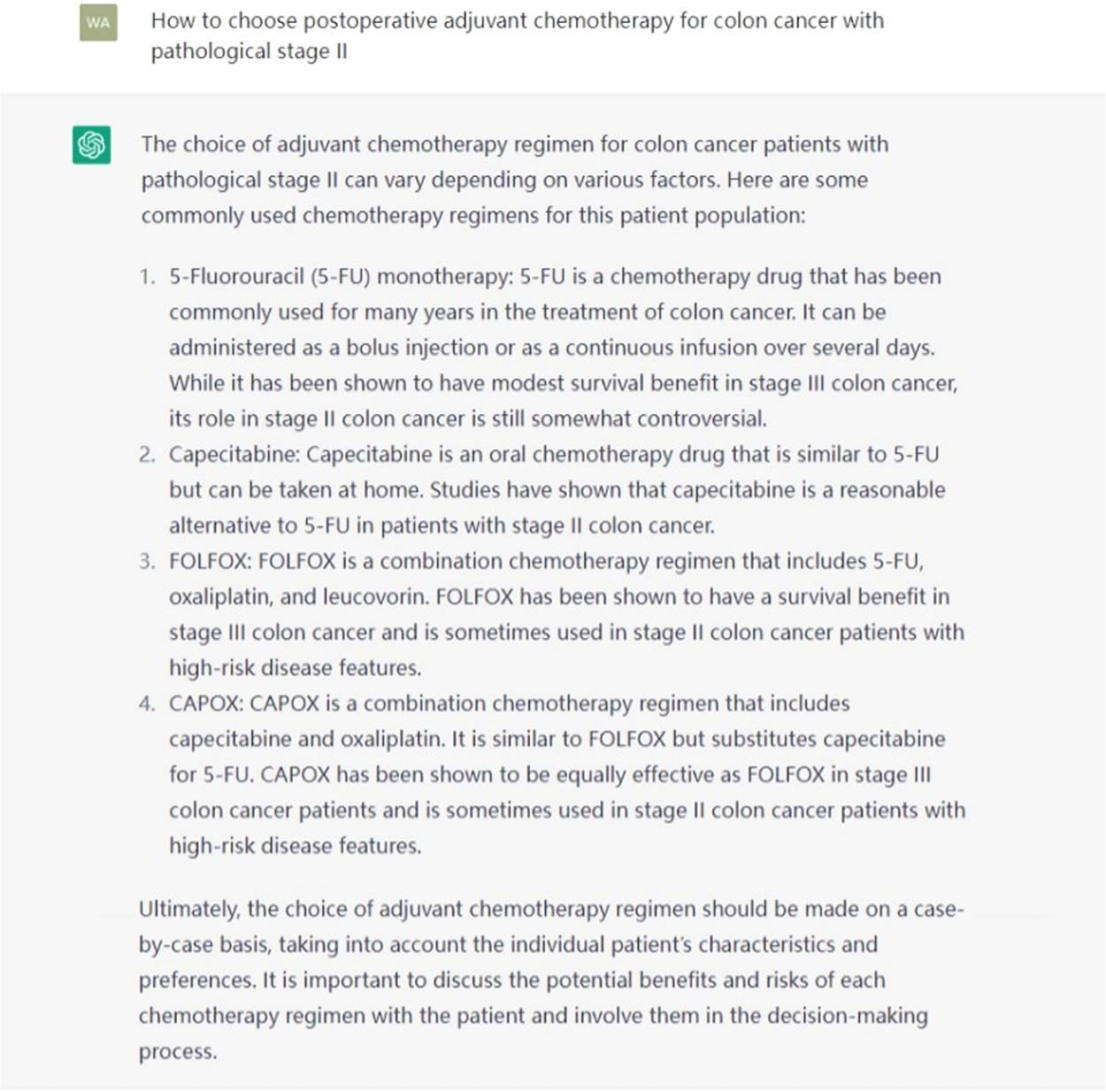
The first time asked ChatGPT about adjuvant chemotherapy for stage 2 colon cancer.

**Figure 2:**
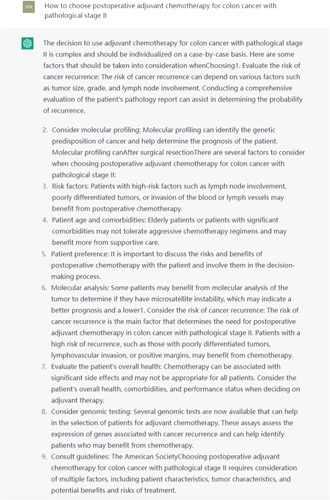
After multiple reminders, I asked ChatGPT again about adjuvant chemotherapy for stage 2 colon cancer.

In summary, the advantage of ChatGPT is that it can be trained, and its disadvantage is that it can also be trained. If someone with ulterior motives uses a data bomb to train unregulated ChatGPT, it will successfully induce ChatGPT to provide answers to its commercial characteristics. If a large amount of data are used for debugging and training, coupled with the rapid development of AI technology, ChatGPT has the potential to become a powerful medical assistant for humans in the near future^3^. This is one of the important reasons why we should always maintain a clear-headed and cautious attitude when using ChatGPT.

## Ethical approval

Not applicable.

## Consent

Not applicable.

## Sources of funding

Not applicable.

## Author contribution

L.S.: devised the concept, performed the literature search, and drafted the letter.

## Conflicts of interest disclosure

The author declares no conflicts of interest.

## Research registration unique identifying number (UIN)

Not applicable.

## Guarantor

Not applicable.

## Data availability statement

The template data collection forms are available from the corresponding author (L.S.) upon reasonable request. The correspondence is based exclusively on resources that are publicly available on the internet and duly cited in the ‘References’ section. No primary data were generated and reported in this manuscript. Therefore, no data have become available to any academic repository.
